# Paramedics’ perceptions of job demands and resources in Finnish emergency medical services: a qualitative study

**DOI:** 10.1186/s12913-022-08856-9

**Published:** 2022-12-02

**Authors:** Christoffer R. Ericsson, Veronica Lindström, Ann Rudman, Hilla Nordquist

**Affiliations:** 1 Faculty of Medicine, University of Helsinki, Helsinki, Finland; 2 Department of Healthcare, Arcada University of Applied Sciences, Helsinki, Finland; 3 Department of Nursing, Umeå University, Umeå, Sweden; 4 Ambulance Service Umeå, Umeå, Sweden; 5 Sophiahemmet University, Stockholm, Sweden; 6 Department of Clinical Neuroscience, Karolinska Institutet, Stockholm, Sweden; 7 Department of Health and Welfare, Dalarna University, Falun, Sweden; 8 South-Eastern Finland University of Applied Sciences, Kotka, Finland; 9 Faculty of Social Sciences, University of Helsinki, Helsinki, Finland

**Keywords:** Emergency medical services, Paramedics, Job demands, Job resources, Qualitative

## Abstract

**Background:**

Paramedics’ fatigue is rising. Stress factors show increased risk for burnout, fatigue, leaving the profession, decreased performance and risk for patient safety. Meanwhile, paramedics’ strong community of practice, autonomy and a sense of professional respect are important factors in forming psychological resilience. We aimed to explore Finnish paramedics’ perceptions of job demands and resources.

**Methods:**

Our study design was descriptive, inductive with a constructivist approach. Using reflexive thematic analysis, we analyse open-ended questions, from a web-based survey and essays written by Finnish paramedic masters-degree students. The study followed the SRQR checklist.

**Results:**

We identified paramedics’ job demands as stress from a high workload, environmental factors and emotional burden. Performance expectations and a sense of inadequacy were further noted, as well as an organizational culture of hardiness, presenting lack of support and sense of inequality. Paramedics’ job resources were pressure management strategies, which were expressed as positive coping mechanisms, agency to affect workload and professional self-actualization, expressed as psychologically safe work community, professional pride and internal drive to professional development.

**Conclusions:**

Finnish paramedics exhibit resources and demands related to uncertainty and emotional burden as well as cultural hardiness and psychological safety in communities.

**Patient or public contribution:**

This study was done based on survey data collected and analysed by the authors. No patient or public contribution was utilized for this study.

## Introduction

Healthcare services are at a breaking point, with personnel fatigue and intent to leave at high levels [1]. An increase in personnel intent to leave is also noticeable among the paramedic profession [2–4]. Although catalysed by healthcare reforms and global pandemic, evidence suggests this is not a novel trend. From a healthcare service perspective, emergency medical services (EMS) is a specific context; paramedics are often highly trained healthcare professionals on the front lines, involving autonomous decision making with limited outside hospital resources while working in unpredictable environments under emotional pressure [5–7]. All the while, paramedics are confronted with a variety of stressful elements [7, 8] or job demands, that is, those aspects of the job, either social, organisational or physical requiring sustained mental or physical effort and leading to increased stress and burnout in their work [9].

A paradigm shift regarding the role of EMS and paramedics, from traditional critical live-saver and transporting towards more specialised healthcare service providers [10], accompanies a certain level of expectations, both societal [10, 11] and from within the profession itself [11]. Consequently, paramedics more often require both broader and deeper competencies to perform their daily clinical work, with wide variations in the complexity of patient encounters [12, 13]. Therefore, an increasing number of paramedic professionals hold a university-level degree [13–15]. Meanwhile, clinical patient situations that paramedics might not necessarily have the tools, knowledge or guidelines to handle often involve patients presenting complex clinical or social problems [16]. This broad responsibility correlates with a sense of helplessness or inadequacy among paramedic professionals [7]. Inadequately fulfilling caring work according to their own ethical standards might also lead paramedics to develop moral distress, a phenomenon often occurring when healthcare professionals feel powerless to take the ethically correct action [17, 18].

Other commonly reported job demands among paramedics are environmental, sociocultural and organisational; critical incidents causing psychological distress [19]; traumatic events concerning patient care [20]; the threat of violence from patients and bystanders [21]; and the risks related to alarm-vehicle driving [22], uncertain working environments [8] and physical injuries [23, 24]. Meanwhile, individual performance pressure [25], conflicts of an ethical nature [26] and social pressures [7], as well as continuously occurring emotional patient encounters [27], seem to be inherent phenomena within the EMS profession. Hence, paramedics’ own emotions are an inherent part of caring work [25]. This—combined with an often underlying organisational culture of stigmatising professionals’ own mental well-being—can lower paramedics’ thresholds to ask for support [28]. Accumulations of such aforementioned stress factors have shown correlations with an increased risk for paramedics’ burnout and fatigue and even the changing profession [5, 27, 29, 30], as well as decreased performance and a higher risk for patient safety [31].

Choosing to take a descriptive view without expectations of providing explanatory perspectives, as we considered the novelty of Finnish paramedics as research population, we set out to investigate how paramedics view their work. With an underlying interest in how it relates to their work well-being, we considered the use of the job demands and resources (JD-R) model as adequate for sorting the data [32, 33]. Developed by Demerouti and Bakker, the JD-R model is a validated theoretical model showing a pathway between job demands and resources to work-related stress, burnout and compassion fatigue [32] and, inversely, positive and fulfilling engagement in work [34, 35]. Although the JD-R model is mainly used in quantitative approaches, it has also been utilised for qualitative applications in attempts to explore healthcare workers’ stress more deeply [33, 36, 37].

The current study aimed to describe paramedic professionals’ work and their own perceptions of job-related demands and resources, patient care and their relations within their professional community. This was accomplished utilising two research questions: 1) What job demands do Finnish paramedics perceive as being related to work in EMS? 2) What job resources do Finnish paramedics perceive as being related to work in EMS?

## Methods

### Study design and theoretical perspectives

With the aim of generating knowledge of individual experiences—that is, forming an enriched and wide understanding of paramedic professionals’ own perceptions of work-related demands and resources by considering patient care, as well as paramedics’ own positions within their professional community—the present study adopted a qualitative descriptive design. This design form attempts to understand phenomena and explore the meanings attributed by participants [38]. Because we strived to understand the socially produced meanings and experiences within the sociocultural context of EMS work, we adopted a constructionist perspective [39, 40]. Further, because the research questions aimed at exploring participants’ feelings, experiences and attributed meaning of their work, an experiential orientation, that is, exploring how the work was subjectively experienced by the participants [39], was adopted as a qualitative framework. Noting the scarcity of previous studies with qualitative approaches [7] and exploring questions on work demands and resources among paramedic professionals, the data were viewed through an explorative rather than confirmatory lens, hence resulting in an inductive approach towards data analysis [39]. The study followed the EQUATOR SRQR (Standards for Reporting Qualitative Research) [41] checklist.

Study trustworthiness was addressed through means based on frameworks noted by Nowell et al. (2017) and following the steps of reflexive thematic analysis, established by Braun and Clarke [42]. For study credibility, the first author engaged in the material for prolonged times, systematically reviewing the data and with time intervals between analyses to allow for the reframing of previous observations [43]. We applied researcher triangulation because all authors participated, at some point, in the analysis phase. Because of the sampling method (web-based survey and student essays), member checking to go over interpretation with the participants was not possible. We describe the process in detail to better enhance dependability, while transferability or generalisability of the study was addressed through descriptions of the context, data collection and participant demographics [43].

### Context: Finnish emergency medical services

From an operative perspective, paramedic work in Finland is divided into two distinct levels: the basic and advanced levels. The latter requires higher competencies involving more autonomy and responsibilities of patient assessment and care. As such, in Finland, although basic-level paramedics hold a degree as practical nurse, registered nurse or firefighter, advanced-level paramedics are required to hold a bachelor’s degree in either nursing, here with an additional advanced-level specialisation course, or a separate bachelor’s degree in emergency care nursing [15, 27]. The standard degree for advanced-level paramedics in Finland is the four-year bachelor’s degree in emergency care (240 ECTS), which is a dual-degree with an integrated bachelor’s degree in nursing. Compared to a nursing degree, the emergency care nursing degree specifically adds competencies in pathophysiology, pharmacology, autonomous patient assessments, clinical decision-making, non-technical skills and acute care treatments as well working as a part of other out-of-hospital authorities. This dual-degree model is unique, with only a handful of countries—mainly Finland, United Kingdom and Australia—offering similar educational models [15]. In addition, paramedic and prehospital-focused master-level degrees are also available; these degrees have gained popularity and traction among professionals. A general social prestige of paramedics in Finland is notable through media portrayals and various public profession appreciation inquiries, which potentially correlates with the high numbers of applicants to paramedic bachelor’s degrees [44], low dropout rates among degree students and the students’ general satisfaction with their degree [45]. Of all the students admitted to emergency care bachelor-level degrees between 2011 and 2021, females have constituted between 55 and 70% [44].

### Data collection and participants

The data consisted of two separate datasets (datasets A and B) to gain multiple perspectives and validate the data. Data were collected at two different time points between 2018 and 2019. The study used a mixed sampling method: targeted snowball sampling for dataset A and purposeful sampling for dataset B. Both datasets were originally written in Finnish (datasets A and B) and partly in Swedish (dataset A) because these are the two official languages in Finland.

Dataset A was collected by the first author in December 2019 through three Finnish social media groups, all of which focused on paramedic and emergency healthcare personnel and that are closed to the general public. Permission to use the groups for data collection was granted from their respective administrators. The collected data from dataset A consisted of two open-ended survey questions: ‘Describe what factors relating to paramedic assignments, the work environment or time off from work have a positive effect on your work well-being as a paramedic’ and ‘Describe what factors relating to paramedic assignments, work environment or time off from work have a negative effect on your work well-being as a paramedic’. The requirement for inclusion was a minimum of 12 months of work experience in EMS operative work, regardless of operative level (basic or advanced level) or degree. This requirement was based on the premise that, prior to 12 months of work experience, some Finnish paramedics might be working under supervision status (supervised by more experienced colleagues). Thus, not being autonomous practitioners might affect their viewpoints. Administrative personnel and volunteer firefighters were excluded because of their low or highly varying levels of operative paramedic work experience.

Dataset B was collected by the last author in fall 2018 and consisted of written essays by Master of Health Care students with a background in emergency care nursing at one University of Applied Sciences in Finland. The essays were written as part of a course independent of this study and utilized due to their range of content, in relation to this study aim. Students taking a master-level degree at the University of Applied Sciences at the time of the study needed to have a minimum of three years of work experience, more often but not exclusively in EMS, before commencing studies. This particular degree programme aimed at paramedic professionals, which enabled a focused, purposeful sample of experienced paramedics.

### Data analysis

Analysis was performed using reflexive thematic analysis, here by following the steps set out by Braun and Clarke [42, 46]. Data analysis was initiated by the first author. Familiarisation of the data was done by the first and last authors and resulted in a general and similar overview and interpretation of the datasets. Both datasets were pooled before initiating the first steps of analysis. This was done to better identify the overlapping themes because the purpose was not to identify the potential differences between the population groups.

Coding was done, for practical and economic reasons, using Microsoft Word 2019 with process spreadsheets constructed in Microsoft Excel 2019. During the preliminary coding phase, all parts of the main texts relating to any identified codes were translated to English by the first author with the aim to harmonise the interpreted data from the start. The coding phase, performed by the first author, was done for both datasets. As recommended by Braun and Clarke [46], a second iterative round of coding followed, resulting in further revision and evolution of the final codes. This was then followed by thematic construction. This phase resulted in six main themes. A review of the initial themes was performed for the primary codes and dataset, first between the relationships ‘data to item-code’ and further between themes and dataset. The themes were all developed from a mixture of both semantic and explicit meanings in the data, as well as latent and underlying interpretations and assumptions. The six main themes were further categorised into either job demands or resources, according to their fit in the construct definitions, that is, whether they were considered motivating and stimulating or stressful and requiring effort [34]. The constructed themes were finally reviewed by all authors before settling on them.

### Reflexivity and researcher characteristics

The term ‘reflexivity’ refers to the researchers’ knowledge of their own role and how it influences their perspectives and interpretation of the subject. Reflexivity involves not merely observing and examining our own practice, but also questioning our ways, epistemological production of knowledge, methodology and ontology [43, 47].

To further test the validity of our findings, we involved multiple investigators in our analysis [48]. Two of the authors have several years of clinical experience within EMS, both deeply acquainted with the role of emergency care or paramedics. In many cases, this aided in a deeper contextual understanding of the participants’ descriptions and formation of the themes and also placed us, at least in part, as insiders in the community of practice. We are also aware that—especially for the first author, for whom this research forms part of a doctoral thesis and who is also strongly connected to the Finnish paramedic field—this perspective carries a risk of bias when it comes to accepting unchallenged and certain assumptions in the data. These biases were accounted for by continuous follow-up with the other authors, some of whom shared more of an outsider perspective, that is, not being in prehospital work. Three of the authors also have extensive experience in research conducted around nursing professionals’ well-being and work stress, which aided in understanding the methodological aspects involved in the formation of the codes and themes. Further, when it comes to the quality of the language translation, the first author has attained an Academic IELTS certificate at the C1 level, which should be considered adequate for text translation.

### Ethical considerations

The study protocol has been reviewed and approved by the appropriate ethical board. All research methods were carried out in accordance with ethical principles and good scientific practice as defined by Finnish National Board on Research Integrity TENK [49]. Informed consent was obtained from all participants prior to commencing data collection via explicitly stated consent check-box (data set A) and through a written consent (data set B).

## Findings

### Participant characteristics

The survey from dataset A generated a response rate of 174 and 164 responses, respectively. The responses of short answers ranging from four to five words to several phrases. Dataset A consisted of 109/182 females (60%), age 31 years (average) with operative work experience of 4.5 years (average), here ranging from 1 to 30 years of experience. Dataset B consisted of 34 written essays, ranging from four to twelve pages per essay, with an open-ended task to reflect solely on one’s own thoughts around paramedics’ work well-being, demands and resources. The study sample of the second datasets consisted of 50% male participants, age around 30 years (average), with all participants having three or more years of work experience as paramedics.

**Fig. 1:**
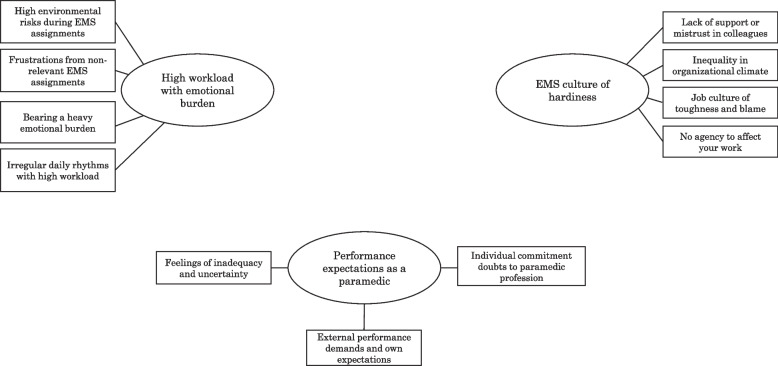
Job demands as percieved by paramedics

**Fig. 2:**
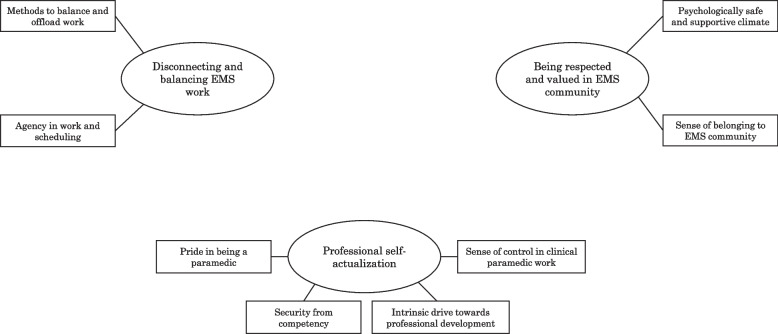
Job resources as percieved by paramedics

The current study developed six main themes: disconnecting and balancing your EMS work, being respected and valued in the EMS community, professional self-actualisation, high workload with emotional burden, EMS culture of hardiness and performance expectations as a paramedic. First three themes were identified as job demands, while the last three themes were identified as job resources. For a visual map of the main themes with their subsequent subthemes, see Figs. 1 and 2. All quotes are marked with a dataset, A or B, and participant number.

### Job as demands perceived by Finnish paramedics

The main themes categorised as paramedics’ job demands were high workload with emotional burden, EMS culture of hardiness and performance expectations as a paramedic.

#### High workload and emotional burden

The theme of ‘high workload with emotional burden’ was described richly in different forms, connecting to both environmental hazards and personal emotional levels. Within this theme, the paramedics described the environmental risks during EMS assignments, most prominently focusing on paramedic-directed violence and aggressiveness from patients and bystanders, faulty or non-functioning equipment and the risks related to road traffic accident scenes and personnel safety during emergency response driving. As one participant stated; ‘Driving to each emergency response has an overall increased risk of accident compared with normal traffic’ (A-34). Another subtheme developed from this was paramedics’ frustration of nonrelevant EMS assignments, here stemming from both systemic healthcare problems relating to dispatch systems and hospital centralisations and paramedics’ more personal sense of ‘increased helplessness’ among patients, which manifested in a workload often deemed as unnecessary. The paramedics also described bearing heavy emotional burdens, which were more often connected to paediatric trauma and resuscitations, along with witnessing elderly loneliness and displacement. One participant emphasised that ‘the sense that seeing all the grief, bad living conditions, alcohol use and displacement and family problems had a bigger impact than any other part of EMS work’ (A-18), as well as traumatic deaths and taking part in the palpable grief and sorrow of relatives. Irregular work rhythms with high workloads were mainly connected to a lack of rest and sleep, mostly because of the high prevalence of night-time assignments. Some also mentioned additional work that was added on top of the operative on-call work.

#### EMS culture of hardiness

The theme of an ‘EMS culture of hardiness’ was described by the paramedics as constituting an unsupportive organisational climate, here described as a lack of appreciation on both the manager and organisational levels, a sense of inequality in leadership and a lack of mentoring. Also, a lack of support from—or even mistrust in—paramedic colleagues was related to a sense of having to deal with partners who were either deemed incompetent or who showed generally bad attitudes and behaviour towards patient care, which the participants described as stressful and as them having to change partners too often. The paramedics also identified job cultures valuing toughness and blaming attitude as contributing to negative climates and increasing stress. These cultural attitudes were strongly related to a stigmatisation of mental well-being and high threshold for using—or entirely lacking—critical event defusing systems, noting that ‘the threshold to activate defusing is not always used because of a prevailing “macho” culture’ (A-5). Further, some mentioned favouring dysfunctional coping methods, such as excessive alcohol use and having bad role models, as well as a general lack of introspection in what was contributing to their decreased motivation and well-being. As one participant stated, ‘You sometimes bump into experienced cynical paramedics with a hardened “nothing affects me” attitude, which might have bad influences of younger, inexperienced colleagues and raise the threshold for defusing need’ (A-4). Furthermore, the paramedics also described a lack of agency when it came to controlling their own work through shift rotations, stationing and work schedules, thus negatively affecting work–life balance and stability in work conditions.

#### Performance expectations

The theme of ‘performance expectations as a paramedic’ was described both from individual perspectives and from the organisational and societal expectations of EMS. The paramedics described feelings of inadequacy and uncertainty, mostly relating to patient care. One participant exemplified ‘situations where you feel that you can’t help the patient as much as you want, like arranging for homecare’; (B-82) while another participant, related to competency requirements, noted; ‘Although continuous theory testing strives to uphold knowledge retainment, they become stressful because you need to do them on your own time, and you have no time to learn because of a high operative workload. This leads to uncertainty’; (A-27). Descriptions included paramedics’ limited tools and knowledge to be able to treat patients as well as they wanted to. This was related to assignments of social distress and complex calls, where the patients’ primary problem was not somatically related. Some paramedics also described a lack of support in challenging clinical decision making as contributing to cumulative stress. Furthermore, external performance demands and internal pressures were described when it came to clinical patient care. These descriptions included social stress from meeting patients or relatives in crisis situations and a general expectation of paramedics’ abilities to always be able to perform well under pressure and not make mistakes, while also meeting organisational expectations to perform well in annual competency testing. For example, one participant stated, ‘Seems like paramedics are the most tested professional group in healthcare. There’s basic-level and advanced-level tests once a year, along with medication tests and simulation tests. Preparing for these is off-work time, and for many, this constant testing increases stress’ (A-17). A theme of paramedics’ uncertain commitment to the paramedic profession was also formed, which came from various descriptions of continuous organisational changes, an insecurity of work availability, a lack of career advancement options and mentions of bad pay: ‘Apart from EMS supervisor, there are not many advancement or specialisation options available in EMS’ (B-2).

### Job resources perceived by Finnish paramedics

The themes categorised as paramedics’ job resources were disconnecting and balancing EMS work, being respected and valued within the EMS community and a sense of professional self-actualisation.

#### Balance of work and private life

The theme of ‘disconnecting and balancing EMS work’ included the participants’ descriptions of functional methods to balance and offload their work in EMS, as well as having a sense of agency in their own work, which was most notably related to work scheduling. The main theme was formed based on both direct descriptions relating to the importance of exercising and family support, as well as inferences based on the participants’ descriptions on locus of control, finding solace in religion or philosophy or, at times, professional help. The 24-h work shift, where paramedics are rostered to work 24 h followed by three full days off, was considered beneficial for work–life balance by most, even though there were also signs of favouring shorter, 12-h, shift lengths. One participant summarized that ‘twelve-hour shifts lead to more work travel, irregularity in shifts and consecutive shifts, making work–life balance difficult. In addition, a 12-h shift more often goes into overtime’ (B-35). To recuperate from work, the participants described the importance of disconnecting from work through, among others, physical exercise, going into nature and sleeping and the necessity of family and peer support. Some descriptions also included the compartmentalisation of the professional role and being able to ‘disconnect from personal emotions to shield from feeling the pain’ (A-12). The importance of being able to affect one’s own work schedule and even station placement was raised by the participants as strengthening their job well-being.

#### Respect and value within job community

The theme ‘being respected and valued in EMS community’ was formed from the concepts of a psychologically safe and supportive work climate, as well as a sense of belonging to the EMS community. This theme constituted a notable part of the findings, with descriptions ranging from being able to offload to peers ‘in a good work environment, where you can talk about everything that makes you feel unsure or bad’ (B-31), having low threshold opportunities for informal peer defusing and formal debriefing. One participant noted that while ‘there are defusing systems in use in my workplace, and I find them vital to uphold your mental capacities … The threshold to activate is low, even lower if the voluntary firefighters have been involved’ (A-1). Concurrently, an open, trustworthy and non-judgemental culture was noted as ‘the attitude has moved to a healthier direction; now, it’s more natural to talk about accumulated mental load and emotions related to challenging or shocking calls’ (A-24). This related closely to the participants’ sense of value in being part of an intimate and tight job community, a ‘camaraderie’ where colleagues take care of each other with professionalism, collectivism and ‘respect for each other’s skills and competences and for the profession’ (B-15) being emphasised by some as core values. A common thread in the paramedics’ descriptions conveyed the importance of being seen as an acknowledged and respected member of their EMS community.

#### Self-actualization as paramedic

The theme ‘professional self-actualisation’ was derived from a wide variety of descriptions relating to personal pride in being a paramedic, having a sense of safety in their clinical competence and, thus, sense of control in clinical work, as well as an intrinsic drive to further their professional development. The participants described the feelings of success and accomplishment in being able to help others through ‘good patient contact, either as a successful intervention, or just generally a good contact, makes you feel good’ (B-40) and sense of privilege from doing their paramedic work as being a force of good, such as ‘when you save a teenager in a motocross accident, resuscitate a baby or notice that the alcoholic patient has acute pancreatitis so that you can initiate treatment on the scene’ (A-23). This was related to descriptions of high self-confidence in making decisions, stemming from both the paramedics’ education, continuous professional training and having the tools to help. On a more detailed level, the utilisation of in situ simulation-based learning to build a toolbox for handling difficult and complex situations was raised as helping them strengthen competence. Overall, some of the participants described a sense of security resulting from competence. As one participant put it; ‘Knowing you’ve done the best you can’ (A-27). As a result of EMS being ‘a challenging and wide field with increasing competency requirements in medicine and care’ (A-22), many of the participants felt an internal will to develop both personally as a paramedic and their working community. A need for constructive and positive feedback from patients, paramedic peers and supervisors were raised as a necessity for achieving this goal.

## Discussion

Our aim was to describe what Finnish paramedic professionals perceive as job-related demands and resources in their EMS work. Our findings identified both paramedics’ own emotional responses and the environmental and organisational factors, some potentially anchored in deep cultural values around managing and performing. Although previous research by Lawn et al. [7] and Afshari et al. [8] supports our findings of paramedics’ job demands, we also identified novel themes. Cultural values were commonly related to Finnish paramedics’ strong sense of role expectations and a pressure to continuously perform well, which was coupled with recurring implications of prevalent strong ‘macho’ cultural values within the organisations; these values had potentially negative effects on individuals’ threshold to address mental well-being issues.

One of the traditionally rooted aspects of paramedic work is having wide clinical knowledge and emergency care skill sets with expectations to perform under various demanding situations [8], often during 12- to 24-h long shifts, when both mental and cognitive capabilities might vary considerably. Paramedics further need to have the ability to function under diverse circumstances while still being adaptable to changes, both regarding the environments they work in and their patients’ acutely deteriorating conditions, but also in handling their own—as well as others—emotional responses. Henckes and Nurok (2015) note that emotion work in EMS is multifaceted, often involving paramedics having to continuously adjust their own emotions to that of their partners, while having limited time for necessary reflection or rectification [25]. This expectation was also noted in our results, with the participants’ expressions relating to the emotional dynamics between job partners, which could sometimes be perceived as burdensome, especially during long 24 h shifts, if mutual trust or an ethical value base was not present.

As noted in our findings and further supported by previous work by Lawn et al. (2020), paramedics’ job demands can be both externally formed, such as how their work is organised and supported or more private in nature, which can involve facing adversities or lack of control over their work [7]. Our study mirrored this, with findings suggesting Finnish paramedics’ performance expectations seemingly are derived from a motivation to perform to high standards that are partly catalysed by the previously mentioned organisational and cultural norms. The Finnish paramedics expressed having an adequately high level of competency in relation to the work demands, and they also felt that this empowered a sense of control over their clinical work, giving them the ability to handle most situations. Such expectations of performance, however, may form a dual-edged sword; for some, this may catalyse a personal drive towards developing their competencies further and moving towards professional self-actualisation [50], which is a positive outcome also presented in our research. Meanwhile, others may consider such expectations as adding stress to their work [8]. This manifested, among others, in the form of annual formalised competency tests, which some attributed to increasing their work-related stress, as did feelings of having to live up to the role of a highly educated paramedic. Such job cultural expectations also pertained to the paramedics’ sense of having to manage a continuing high mental workload, potentially accumulated during 12- or 24-h shift with concurrent calls.

Furthermore, EMS work involves inherent environmental hazards and risks that need to be managed, some on an almost daily basis. Notable environmental or assignment-related hazards are an increased prevalence of violence and threats towards paramedics [8], which has reached a point where it might be expected and even normalised [51]. Other risks we discovered relate to road scene safety and increased risks during alarm-vehicle driving [22]. This potential for risk situations is clearly categorised as demands adding to paramedics’ daily job stress and mental workloads.

Paramedic work inherently involves job-related resources, that is, aspects that are functional for work achievement, motivating and stimulating personal development or reducing job demands [34] and, hence, leading to higher job engagement. These resources include strong peer communities of practice [25], a sense of autonomy and reported high levels of social prestige of the EMS profession [52]. Such sociocultural and organisational aspects have been reported as mitigating negative stress outcomes in acute caring work [25] and, as presented in a systematic review by Yu et al. (2019), are important factors in the formation of resilience among acute healthcare personnel [53]. Notably, EMS seems to form a respected community of practice, connecting to a strong sense of belonging among paramedics [25]. This might stem from spending such long shifts, often between 12 to 24 h, together. As also noted in our findings, a strong sense of professional identity seemed to shape personal pride in being a paramedic [54]. These may well function as job-related resources towards thriving at work, especially if combined with a high degree of support and psychological safety within the job community [55]. These findings are also mirrored in our results, supporting the notion that many participants felt an almost familial bond with their paramedic peers and that they could openly discuss their own emotions and uncertainties within their community.

A concurrent aspect also evident in our findings was paramedics’ expressions of having to bear an emotional burden stemming from encounters with patients and relatives. Based on paramedics’ own perceptions, such emotions traditionally seem to be commonly related to experiences involving acutely sick children and lonely elderly. Although these clearly differ from each other, a potentially common denominator between such experiences might be paramedics’ sense of helplessness; paediatric patients are generally rare in EMS and often elicit stronger emotions in healthcare personnel [56], here buoyed by feelings of fear and, perhaps, uncertainty [57]. Meanwhile, daily occurring EMS assignments involving patients with complex pathologies or social distress, such as elderly loneliness, isolation, long-term substance abuse or chronic conditions, are not easily managed and treated before arriving at the hospital. As noted earlier, although the competencies that paramedics had built during their training were deemed adequate and relevant for the actual practical work, which many also expressed provided a sense of safety in their clinical work, paramedics are inherently more specialised in treating emergency conditions and trauma care and, as such, are not always equipped to handle such aforementioned situations, which often fall outside their range of specialties [11]. As our findings suggest, this might connect to a sense of inadequacy, mainly from not being able to help as much as one would want [7]. This has the potential to manifest as paramedics’ general frustration over ‘nonrelevant’ or even ‘nonsensical’ assignments, especially as such assignments may easily be a majority during 12- or 24-h shift, where chances to mentally recuperate between them might be low. Mausz et al. (2022) have also noted this form of cognitive dissonance as stemming from the discrepancy in paramedics’ expected role and what turns into an espoused paramedic identity, manifesting in such frustrations of nonurgent calls and frequent ‘system abusers’ [58]. Similar phenomena have been noted by Lazarsfeld-Jensen (2014), who investigate how storytelling and rescue myths imbued on young graduate paramedics might enforce an image of paramedics as rescuers or ‘masters of chaotic spaces’, working mostly as live-savers and ‘requiring critical events as proving grounds’ [11].

Although there is ample evidence of paramedics’ occupational stress factors and their subsequent effects, there is a paucity of specifically qualitative literature aimed at exploring paramedics’ individual perceptions of what they identify as occupational demands and resources, something that is also noted in a systematic review by Lawn et al. [7]. Such a bilateral perspective is also relevant for identifying the underlying aspects related to paramedics’ stress levels and understanding their work engagement and individual resilience development while also considering the need to develop organisational psychosocial safety and educational support.

Our aforementioned findings on expectations and managing form what could be described as the bane of EMS: paramedics seem to form a community of resilient professionals [59], independently functioning frontline emergency clinicians who are expected to hold large amounts of knowledge and skills but who also are expected to carry the emotional burden of their work on their own shoulders. The undercurrents of a prevailing culture of hardiness, described also in our findings as a ‘macho culture’, were evident within the Finnish paramedic communities. This manifested as a lack of proper peer and manager support, lacking psychological safety, high thresholds to activate defusing systems and paramedics’ sense of general inequality in relation to management, lacking the feedback or possibilities for career advancement. As noted by Lawn et al. (2020), asking for support within a culture of hardiness might be seen as a sign of weakness or, worse, not belonging [7]. Although there are signs that such ‘old-time cultures’ are slowly subsiding and being replaced by a more open and psychologically aware mentality, these findings are not geographically unique because they have been noted in EMS cultures in previous research [28].

An often-iterated presumption is that strong mental resources are—or should be—the natural requirements of paramedics and first responders. In other words, they should be resilient. Of course, this implies that a focus on individual resilience is relevant in the first place, something that the inherently unpredictable work in EMS might well justify. However, the notion of having this ‘right stuff’ from the start is simply not feasible. There is certainly an advantage, especially at the early stage of one’s career, in having adopted good and functional coping mechanisms to handle the unexpected job-related stressors inherent in EMS work [19]. However, paramedic education needs to focus on identifying and forming such initial building blocks while the work culture and environment then furthers to support them, aiming to strengthen these internal resources and finally enabling the growth of paramedics’ necessary resilience [53]. To further understand the connection between paramedics’ work stress and mental well-being and to facilitate a discussion on how paramedics’ resilience could be constructed and strengthened, future research could look more specifically at the role of job demands, control and support models [60].

### Methodological considerations

The use of Job Demands and Resource model was based on strong validation from within the occupational health literature [32, 33]. This also brings a methodological limitation, as a model is always merely a codified representation of reality and other models could also have been equally suitable. However, we did find that for the scope of this particular study aim, the JD-R model was sufficiently relevant in adding value. The use of the JD-R model from a qualitative perspective further brings challenges, as the model is mainly used for quantitative use.

Our research data represented both a widely dispersed and presumably heterogeneous population and, similarly, a more narrowly focused population consisting of emergency medical care master’s degree students. Although we agree that using students as participants does raise relevant questions regarding power imbalance, as the essays were written prior and independent of the study, as part of a coursework and later, due to their range in content, used for research purposes (with consent), the discrepancy between role as ‘student’ and ‘participant’ becomes more solved. This also gave us the opportunity for an overview on a more general level (data set A) while also examining a smaller sample that presumably represented individuals with internal motivations to develop and broaden their competencies by attaining a higher degree (data set B). A mixed sampling of participants allowed for a balance between similarities and differences in participant demographics and increased the dispersion of their geographical location. We are, meanwhile, mindful of factors which might shape the reporting of participants’ experiences from both data sets, such as, among others, time of day, previous traumatic experiences, recurring memories and/or overall experience of the field as well as ability to verbalize own perceptions and reflections. This was noted in high variability of response lengths in data set A.

Although purposeful sampling aims to select individuals who are especially knowledgeable about a phenomenon of interest [61], targeted snowball sampling through social media is a recognised and viable way to reach a wider public, even though there are known challenges relating to potential bias [62]. Use of web-based survey for qualitative data collection is not ideal compared to focus groups or interviews, which would probably yield more deep and rich qualitative data. However, considering the novel nature of the research field, this method allowed us to cast a wide net, enabling us to capture surface elements which can be explored later in more depth. As the study aim was rather broad, we found using a larger participant count, increased the diversity of experiences from a larger sample [63]. We further agree that, as a qualitative approach, both data sets were exceptionally large and quality was partly shallow (mainly data set A). Although thematic saturation was not addressed explicitly in the analysis, we felt the combination of the distinct data sets, with breadth and depth, impacted the quality of the dialog and thus overall information power [63]. Patient or Public Involvement (PPI) could also potentially yield rich in-depth data. PPI was, however, not utilized in this study due to practical and economic reasons mostly concerning recruitment and timing. However, PPI use in further studies of the subject would certainly add value and would be highly encouraged.

We chose to translate the data from the original languages (Finnish or Swedish) to English. Being aware that language translation always brings the risk of potentially losing nuances or latent implications inherent within the original language used [64], we considered that a common language would still further a more coherent analysis phase. We were also mindful that because the translation was done by the first author, who was familiar with the paramedic and EMS context, certain themes and implications pertaining specifically to the profession would perhaps more easily be comprehended.

## Conclusion

Our research aimed to better understand Finnish paramedics’ expressions of their job resources and demands in EMS work. The paramedics expressed both resources and demands connected to the challenges of daily clinical work, decision making and environmental hazards. Personal role expectations and the underlying stigmatising EMS cultures were also noted as demands. To understand the connection between paramedics’ work and mental well-being and to facilitate a discussion on how paramedics’ resilience could be constructed and strengthened, future research could utilise job demands, control and support models to explore explanatory pathways that EMS work has on the development of paramedics’ psychological resilience.

### Relevance to paramedic healthcare services

Paramedic role has shifted gradually towards being first-line healthcare providers, increasingly meeting and assessing non-acute patients, more often outside their speciality. This might manifest as a sense of helplessness in paramedics, often lacking tools to care for such patients. Furthermore, EMS is a highly respected field although the job culture still shows signs of hardiness and performance expectations, which have to be addressed both early in education and work environments to ensure paramedics’ feel supported in their role and work.

## Data Availability

The datasets generated during and analysed during the current study are not publicly available due to restrictions e.g. their containing information that could compromise the privacy of research participants (i.e. language of data, identifiable factors or specific geographical data) but are available from the corresponding author on reasonable request.
